# Genome-Wide Association Study on Seedling Phenotypic Traits of Wheat under Different Nitrogen Conditions

**DOI:** 10.3390/plants12234050

**Published:** 2023-12-01

**Authors:** Chenchen Hu, Jinghui Li, Jiajia Liu, Dazhong Zhang, Liqiao Jin, Nian Yang, Bipo Bai, Zenghao Wang, Suwei Feng, Zhengang Ru, Tiezhu Hu

**Affiliations:** Henan Provincial Key Laboratory of Hybrid Wheat, Henan Institute of Science and Technology, Collaborative Innovation Center of Modern Biological Breeding, Xinxiang 453003, China; 13569879750@163.com (C.H.); 17746504354@126.com (J.L.); ljjzl2014@163.com (J.L.); zdz1727697945@163.com (D.Z.); myjinliqiao@163.com (L.J.); 15936095936@163.com (N.Y.); 15936575680@163.com (B.B.); 13072631017@163.com (Z.W.); fsw@hist.edu.cn (S.F.); rzg4199@163.com (Z.R.)

**Keywords:** wheat, seedling biomass, root traits, GWAS, nitrogen stress

## Abstract

Nitrogen fertilizer input is the main determinant of wheat yield, and heavy nitrogen fertilizer application causes serious environmental pollution. It is important to understand the genetic response mechanism of wheat to nitrogen and select wheat germplasm with high nitrogen efficiency. In this study, 204 wheat species were used to conduct genome-wide association analysis. Nine phenotypic characteristics were obtained at the seedling stage in hydroponic cultures under low-, normal, and high-nitrogen conditions. A total of 765 significant loci were detected, including 438, 261, and 408 single nucleotide polymorphisms (SNPs) associated with high-, normal, and low-nitrogen conditions, respectively. Among these, 14 SNPs were identified under three conditions, for example, *AX-10887638* and *AX-94875830*, which control shoot length and root–shoot ratio on chromosomes 6A and 6D, respectively. Additionally, 39 SNPs were pleiotropic for multiple traits. Further functional analysis of the genes near the 39 SNPs shows that some candidate genes play key roles in encoding proteins/enzymes, such as transporters, hydrolases, peroxidases, glycosyltransferases, oxidoreductases, acyltransferases, disease-resistant proteins, ubiquitin ligases, and sucrose synthetases. Our results can potentially be used to develop low-nitrogen-tolerant species using marker-assisted selection and provide a theoretical basis for breeding efficient nitrogen-using wheat species.

## 1. Introduction

Wheat (*Triticum aestivum* L.) is a pivotal crop in global agriculture. It is widely cultivated and plays an indispensable role in human food production [1], and its yield is largely influenced by fertilizer application, irrigation, and climate [2,3]. During the wheat growth process, nitrogen (N) is a crucial nutrient that exists in both inorganic forms (such as nitrate and ammonium nitrate) and organic forms (such as amino acids and urea). Nitrate serves as the primary N source utilized in agricultural production [4]. Furthermore, N plays a pivotal role in plant physiology since it constitutes proteins, nucleotides, chlorophyll, and specific plant hormones [5,6], directly impacting cell division and growth.

Insufficient or excessive amounts of N significantly influence root development, aboveground growth, kernel formation, yield and quality [7,8,9,10,11]. Low-N (LN) conditions can stimulate root growth to increase the uptake of N. Earlier studies revealed that the limited availability of N induced the lengthening of crops root and lateral roots [12,13]. Moreover, the changes in the total root length [i.e., ratio of the total root length under LN and high-N (HN) conditions] and shoot N contents are significantly positively correlated under LN conditions [14]. In China, a 71% increase in total annual grain production from 283 to 484 MT (million tons) from 1977 to 2005 was achieved at the cost of a 271% increase in synthetic N fertilizer application (from 7.07 to 26.21 MT) over the same period [15]. Excessive application of N fertilizers has led to the growing prominence of environmental issues, including soil acidification in farmlands and elevated nitrate levels in groundwater [15,16,17,18,19,20]. Therefore, reducing N fertilizer input and improving crop nitrogen-use efficiency (NUE) have attracted more and more attention [21,22].

NUE in wheat is affected by many factors, including genes, regulatory networks, signal transduction, and metabolic pathways [23,24,25]. Meanwhile, the response of wheat to the N environment phenotype depends on stress time, intensity, and genotype differences between species [22,26]. These genotypic differences also provide a basis for breeding genotypes with high NUE [22,27]. With rapid improvements in molecular biology technologies, the genetic basis of crop species with high nutrient absorption and utilization has attracted extensive attention [28]. Genome-wide association studies (GWASs) have the advantages of high efficiency, large flux, strong applicability, and high coverage of genetic diversity, and have become some of the most important methods for gene mining of plant quantitative traits [29]. In recent years, GWASs have been widely used in wheat, soybean, corn, rice, and other crops [30,31,32,33], especially in the genetic mechanism of nitrogen-use efficiency.

Wang et al. used a hydroponic system to analyze the natural variation in 22 root traits and 6 stem traits in 213 maize inbred lines under NN and LN conditions. Through GWAS, 297 significant single nucleotide polymorphisms (SNPs) were detected at different N levels and 51 candidate genes with amino acid variations in coding regions or differentially expressed were identified under LN conditions. Subsequently, researchers re-sequenced the candidate gene *ZmNAC36* and found that 33 variable loci in the promoter region were significantly correlated with the four root traits under LN conditions [34]. Schmidt et al. identified candidate genes involved in stress signaling and hormone regulation in the MAGIC wheat population *WM-800* under LN stress, such as *Rht* (*Rht -B1* and *Rht-D1*) and *NRT1.1* [35]. Fu et al. identified four candidate genes for maize seedling root traits using GWAS, including *Zm00001d004123* (controlling crown number), *Zm00001d051083* (controlling root length and area), *Zm00001d025554* (controlling plant height), and *Zm00001d050798* (controlling total root length). These genes were related to lactoylglutathione lyase, leucine-rich repeat receptor-like serine/threonine protein kinase, aspartic protease synthesis, and E3 ubiquitin protein ligase EL5 synthesis [36]. In particular, many N-efficient genes (such as *OsTCP19*, *OsNAC42*, *OsNPF61*, *OsNLP4*, *OsNiR*, etc.) were identified based on GWASs in rice. Therefore, GWASs can help to improve our understanding of the molecular basis of traits related to N-efficient species and lay a foundation for breeding such species.

At present, the overapplication of N fertilizer in agricultural production has become a common phenomenon, but most of the current studies have been conducted under LN and normal N (NN) conditions [34,36,37,38]. In this study, a total of 204 wheat germplasm resources were studied under three N conditions (LN, NN, and HN). A 55K wheat iSelect SNP array was used for genotype analysis. This study aimed to examine wheat adaptability under LN, NN, and HN conditions and the mining of important SNP sites. Our results will benefit wheat breeding programs and sustainable agriculture.

## 2. Results

### 2.1. Trait Phenotyping

**Figure 1:**
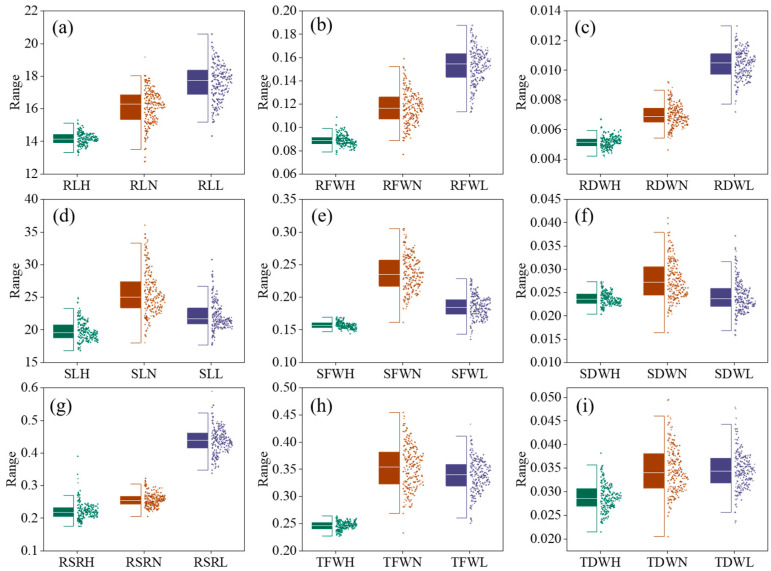
Distribution of BLUP values of nine phenotypic traits in related populations. (**a**) Distribution of BLUP values of RL in related populations; (**b**) Distribution of BLUP values of RFW in related populations; (**c**) Distribution of BLUP values of RDW in related populations; (**d**) Distribution of BLUP values of SL in related populations; (**e**) Distribution of BLUP values of SFW in related populations; (**f**) Distribution of BLUP values of SDW in related populations; (**g**) Distribution of BLUP values of RSR in related populations; (**h**) Distribution of BLUP values of TFW in related populations; (**i**) Distribution of BLUP values of TDW in related populations; Trait abbreviation: RLH, root length in high nitrogen; RLN, root length in normal nitrogen; RLL, root length in low nitrogen; RFWH, root fresh weight in high nitrogen; RFWN, root fresh weight in normal nitrogen; RFWL, root fresh weight in low nitrogen; RDWH, root dry weight in high nitrogen; RDWN, root dry weight in normal nitrogen; RDWL, root dry weight in low nitrogen; SLH, shoot length in high nitrogen; SLN, shoot length in normal nitrogen; SLL, shoot length in low nitrogen; SFWH, shoot fresh weight in high nitrogen; SFWN, shoot fresh weight in normal nitrogen; SFWL, shoot fresh weight in low nitrogen; SDWH, shoot dry weight in high nitrogen; SDWN, shoot dry weight in normal nitrogen; SDWL, shoot dry weight in low nitrogen; RSRH, root–shoot ratio in high nitrogen; RSRN, root–shoot ratio in normal nitrogen; RSRL, root–shoot ratio in low nitrogen; TFWH, total fresh weight in high nitrogen; TFWN, total fresh weight in normal nitrogen; TFWL, total fresh weight in low nitrogen; TDWH, total dry weight in high nitrogen; TDWN, total dry weight in normal nitrogen; TDWL, total dry weight in low nitrogen.

The variation in the phenotypic traits of the 204 wheat accessions under the three conditions was extensive and showed a normal distribution. Nine phenotypic traits were measured and best linear unbiased prediction (BLUP) values were calculated in HN, LN, and NN conditions (Figure 1 and Table 1). Compared to NN, the means of shoot length (SL), shoot dry weight (SDW), and shoot fresh weight (SFW) were lower in the HN condition and LN conditions (Figure 1d–f). However, the mean root-related traits were higher in LN than in HN or NN conditions (Figure 1a–c,g).

The coefficient of variation was between 2.56% and 24.84%. In the HN condition, the mean RL, RSR, RDW, RFW, SL, SDW, SFW, TDW, and TFW decreased by 11.52%, 13.28%, 25.71%, 23.87%, 22.97%, 31.70%, 33.54%, 17.00%, and 30.35%, respectively, compared with those under NN. The mean root-related indices, RL, RDW, and RFW in the LN condition increased by 9.76%, 48.57%, and 31.31%, respectively, compared with those under NN. The average SL, SDW, SFW, and TFW decreased by 13.49%, 30.26%, 21.80%, and 4.24%, respectively. In conclusion, the HN/LN ratio significantly affected the phenotypic characteristics of wheat. The generalized heritability (*h*^2^) ranges of the nine phenotypic traits were from 0.135 (SDW) to 0.92 (RL), from 0.327 (TDW) to 0.79 (RL), and from 0.381 (SDW) to 0.92 (RL) in the LN, NN, and HN conditions, respectively. However, the *h*^2^ of RL in the NN condition was lower (0.79) than that of the other two conditions, indicating a significant environmental effect on RL (Table 1).

**Figure 2:**
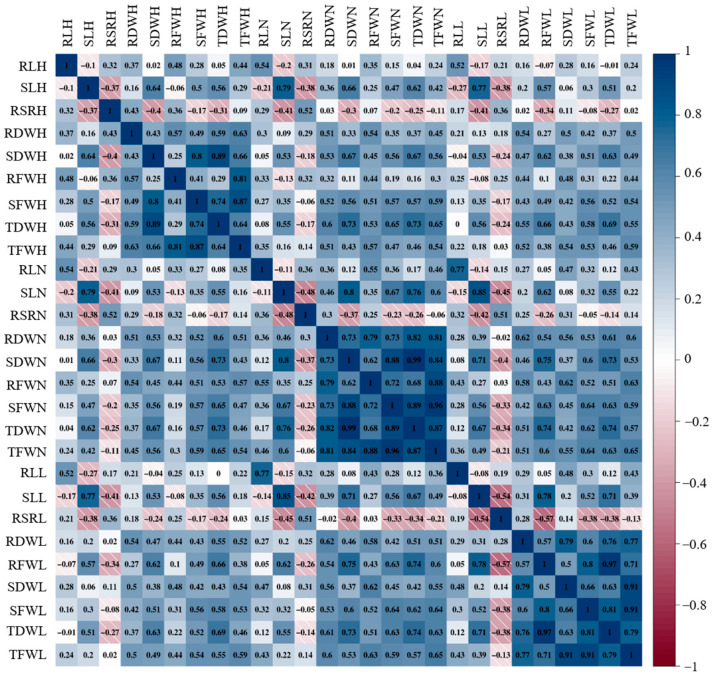
Correlation analysis of nine phenotypic traits for each treatment condition. Trait abbreviation: RLH, root length in high nitrogen; RLN, root length in normal nitrogen; RLL, root length in low nitrogen; RFWH, root fresh weight in high nitrogen; RFWN, root fresh weight in normal nitrogen; RFWL, root fresh weight in low nitrogen; RDWH, root dry weight in high nitrogen; RDWN, root dry weight in normal nitrogen; RDWL, root dry weight in low nitrogen; SLH, shoot length in high nitrogen; SLN, shoot length in normal nitrogen; SLL, shoot length in low nitrogen; SFWH, shoot fresh weight in high nitrogen; SFWN, shoot fresh weight in normal nitrogen; SFWL, shoot fresh weight in low nitrogen; SDWH, shoot dry weight in high nitrogen; SDWN, shoot dry weight in normal nitrogen; SDWL, shoot dry weight in low nitrogen; TFWH, total fresh weight in high nitrogen; TFWN, total fresh weight in normal nitrogen; TFWL, total fresh weight in low nitrogen; TDWH, total dry weight in high nitrogen; TDWN, total dry weight in normal nitrogen; TDWL, total dry weight in low nitrogen; RSRH, root–shoot ratio in high nitrogen; RSRN, root–shoot ratio in normal nitrogen; RSRL, root–shoot ratio in low nitrogen.

The relationship between shoot and root systems was greatly affected by these conditions (Figure 2). Strong correlations were observed among most traits under different N conditions. In the HN condition, SDWH was positively correlated with SFWH and TDWH, with correlation coefficients of 0.80 and 0.89, respectively. In the NN condition, the TFWN was positively correlated with RDWN (0.81), SDWN (0.84), RFWN (0.88), SFWN (0.96), and TDWN (0.87). TFWL was strongly correlated with RFWL (0.91) and SFWL (0.91) in the LN condition. Notably, the correlation between these phenotypic traits in the NN condition were more stable than those in the HN and LN conditions.

### 2.2. SNP Distribution, Linkage Disequilibrium, and Population Structure

**Figure 3:**
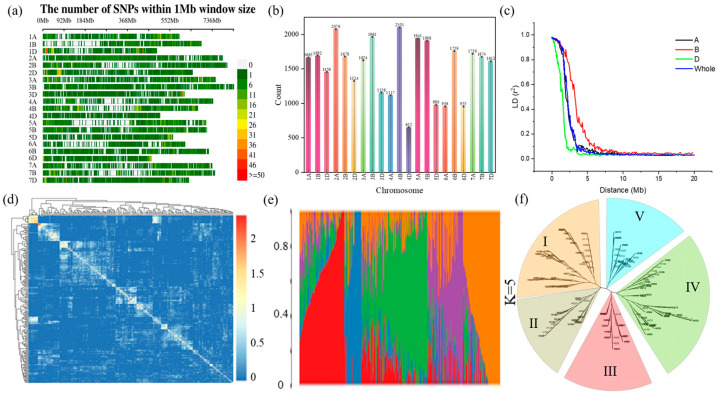
Single nucleotide polymorphism (SNP) correlation. (**a**) Distribution of SNP markers; (**b**) Distribution of SNP markers on 21 chromosomes; (**c**) Linkage disequilibrium (LD) decay diagram. LD refers to the non-random association of alleles at different loci in a population; (**d**) Relationship analysis of associated populations; (**e**) Structural analysis of associated populations represent five distinct subpopulations; (**f**) Dendrogram of neighbor-joining clustering constructed using 32,008 SNPs and 204 wheat species.

After quality control, the 55 K Wheat iSelect SNP array with 32,008 SNP markers was selected for association mapping (Figure 3a). These SNPs were distributed on all 21 wheat chromosomes, with 11,098, 12,775, and 8135 SNPs on chromosomes A, B, and D accounting for 34.7%, 39.9%, and 25.4%, respectively. The distribution of polymorphic loci on different chromosomes was significantly different, ranging from 652 (Chr 4D) to 2101 (Chr 4B) (Figure 3b). With an increase in physical distance, the LD between SNP marker pairs shows a significant decreasing trend. When the truncation threshold of r^2^ was 0.1, the LD attenuation distance of the D genome was the smallest (2.5 Mb) compared to that of the A (4.6 Mb) and B (3.5 Mb) genomes. The LD attenuation distance for the whole genome was 3.5 Mb (Figure 3c). Different methods were used to analyze the population structure. The neighbor-joining method based on Nei’s standard genetic distance was used to classify the 204 wheat accessions, and it indicated that they were divided into five subpopulations (I, II, III, IV, and V). Subpopulation I contains the most varieties (lines), mainly Hebei and Yunnan varieties (lines); subpopulation II is mainly from Xinjiang and Henan; subpopulation III has the least material, mainly including imported varieties (lines); subgroup IV mainly includes some wheat materials from Sichuan and Yunnan; and subgroup V mainly includes wheat varieties (lines) from Henan and Yunnan as well as individual Guangdong (Figure 3d–f).

A total of 765 SNPs distributed on 21 chromosomes were identified for RL, SL, RDW, RFW, SFW, SDW, TDW, TFW, and RSR, explaining 5.88–22.31% of phenotypic variation (Table S2). Genome B had the largest number of SNPs (309), followed by genomes A (273) and D (183). Additionally, 151, 392, and 222 significant SNPs were only identified in the NN, HN, and LN conditions (Table S2).

### 2.3. GWAS of Root-Related Traits

In total, 27, 159, and 116 SNP loci were significantly correlated with RL, RFW, and RDW, respectively (Table S2 and Figure S1).

The SNPs significantly correlated with RL were mainly distributed in 1B, 4B, 6D, and 7D (Table S2 and Figure S1A). In the HN condition, three consecutive SNPs were detected on 1B, concentrated around 666.06–666.57 Mb; these three SNPs could explain 7.41–9.80% of phenotypic variation. In the NN condition, there were three SNPs in the 462.55–463.55 Mb interval on chromosome 6D, which could explain 5.88–8.22% of phenotypic variation. Three SNPs from 627.35 to 628.13 Mb on chromosome 7D could explain 11.16–12.01% of phenotypic variation.

The loci associated with RFW were distributed on the remaining chromosomes, except chromosome 2A (Table S2 and Figure S1B). In the HN condition, five consecutive SNPs were detected on 2D, concentrated at 15.74–17.47 Mb, and these five SNPs could explain 6.95–9.80% of phenotypic variation. There was a major quantitative trait locus (QTL) on chromosome 3B, containing 10 significant SNPs, distributed from 521.79–524.53 Mb, and a single SNP could explain 15.01% of phenotypic variation. In addition, some continuous sites also controlled SFW in the HN condition. In the LN condition, there was a major QTL on chromosome 5B, containing 14 significant SNPs, distributed through 513.87–518.30 Mb, and SNP sites could explain 7.01–10.06% of phenotypic variation. Notably, *AX-109966482* on chromosome 5A was detected in both the NN and LN conditions.

In the HN condition, three important RDW-related loci were distributed on chromosomes 1B, 3B, and 6D (Table S2 and Figure S1C). The relevant loci on chromosome 6D were the main QTL, containing 26 significant SNPs concentrated at 467.21–471.91 Mb. A single SNP explained 9.43% of phenotypic variation. In the NN condition, the significant SNP loci were distributed on chromosomes 1A, 1B, and 1D. There were four consecutive significant SNPs located at 21.67–21.81 Mb on chromosome 1D, and single SNPs could explain up to 8.59% of phenotypic variation. The significant SNP loci of LN were all distributed on chromosome 7A, and the relevant loci were distributed at 20.48–23.34 and 722.18–722.23 Mb, which could explain 8.98–9.87% and 7.49–7.60% of phenotypic variation, respectively. Among all the significant SNPs controlling RDW, *AX-109038149* (1B) was detected in both the HN and LN conditions. *AX-108847203* (1A) and *AX-108871687* (6B) were detected in both the NN and LN conditions. *AX-111028996* (1B) was detected in all three conditions.

### 2.4. GWAS of Shoot-Related Traits

A total of 46, 11, and 92 SNPs were significantly correlated with SL, SFW, and SDW, respectively (Table S2 and Figure S2).

The SNPs significantly correlated with SL were mainly located on chromosomes 1A, 1B, and 7B (Table S2 and Figure S2A). Notably, the significant SNP loci located at 567.71–568.00 Mb on 1A in the NN and LN conditions were consistent, and could explain 8.73–8.94% and 7.16–8.20% in the LN and NN condition, respectively. Significant SNP loci located at 611.78–612.33 Mb on 7B were the same in the NN and LN conditions, and 8.06–10.60% and 7.40–8.37% of phenotypic variation could be explained in the LN and NN conditions, respectively. Of all the significantly correlated sites, *AX-111124688* (6A), *AX-111170306* (1A), *AX-111724995* (1A), *AX-108847203* (1A), *AX-109988415* (2A), *AX-109913880* (4B), *AX-110832233* (4D), *AX-89398511* (4D), *AX-108774819* (7B), *AX-109052370* (7B), *AX-109327847* (7B), *AX-110483224* (7B), *AX-110490694* (7B), *AX-111506964* (7B), and *AX-111609105* (7B) could be detected in both the NN and LN conditions. Two SNPs, *AX-94550983* (1B) and *AX-109627540* (7D), were detected in both the HN and NN conditions. *AX-108876381* (6A) was detected in all three conditions.

SNPs significantly related to SFW were mainly found in the LN condition (Table S2 and Figure S2B), and the main QTL of the significant SNP sites was found at 59.15–62.40 Mb on chromosome 2D. A single SNP could explain 9.16% of phenotypic variation. Additionally, significant sites at 10.79–10.97 Mb responded to both HN and NN conditions.

For SDW, most of the loci detected in the LN condition were distributed on 2D, 4D, 5D, and 6B (Table S2 and Figure S2C). Among these, the main QTLs of the significant SNP sites were found at 59.15–62.40 Mb on chromosome 2D; a single SNP could explain 8.98% of the phenotypic variation. QTLs were also related to SFW under the same conditions. There were eight SNP loci at 114.52–116.46 Mb on chromosome 6B, which could explain 7.59–9.33% of phenotypic variation. Four SNPs at 543.24–543.58 Mb on chromosome 5D could explain 7.47–9.20% of phenotypic variation. In the NN condition, four significant SNPs were found on chromosome 7B at 611.78–612.33 Mb, which could explain 7.20–7.96% of phenotypic variation. Among the significant SNPs, *AX-109326114* (1D), *AX-108962141* (1B), *AX-110832233* (4D), *AX-108847203* (1A), and *AX-111170306* (1A) were detected in both the NN and LN conditions.

### 2.5. GWAS of Biomass and Root–Shoot Ratio

A total of 104, 193, and 195 SNPs significantly correlated with TFW, TDW, and RSR were detected under different conditions (Table S2 and Figure S3).

SNPs that were significantly related to TFW were mainly distributed on chromosomes 5A, 3B, and 7D (Table S2 and Figure S3B). In the HN condition, two consecutive SNPs were detected on chromosome 3B, concentrated at 66.58–66.71 Mb; these two SNPs could explain 7.08–7.50% of phenotypic variation. In the NN condition, six SNPs at 482.68–485.36 Mb on chromosome 5A could explain 8.04–9.25% of phenotypic variation. Five SNPs in the 608.20–610.53 Mb region of chromosome 7D could explain 7.09–8.24% of phenotypic variation. In the LN condition, a major QTL was found on chromosome 5A, containing 11 significant SNPs, from 513.87–517.87 Mb; SNPs could explain 7.34–8.27% of phenotypic variation. Notably, *AX-109815802* (4B) was detected in both the NN and HN conditions.

In terms of TDW, significant SNPs were concentrated on chromosomes 2D and 5D (Table S2 and Figure S3C). In the HN condition, three consecutive SNPs were detected on chromosome 1D, concentrated at 14.71–15.64 Mb; these three SNPs could explain 7.27–7.37% of phenotypic variation. In the LN condition, ten SNPs were found at 59.15–62.40 Mb on chromosome 2D, which could explain 7.28–8.84% of phenotypic variation. There was a major QTL located on chromosome 5D, containing 14 significant SNPs, which were distributed at 543.23–555.04 Mb; these could explain 7.40–9.56% of phenotypic variation. It is worth noting that 20 significant SNPs were found in both conditions; *AX-108962141* (1B) and *AX-110832233* (4D) were found in all three conditions.

SNPs were significantly associated with RSR (Table S2 and Figure S3A). Two important loci were detected in the HN condition and were mainly distributed on chromosomes 1A and 1B. The QTL on chromosome 1A contained seven significant SNPs concentrated at 40.96–41.54 Mb; a single SNP could explain 10.42% of phenotypic variation. Two QTLs were detected on 1B containing 3 and 11 consecutive SNPs distributed at 317.45–317.48 Mb and 322.43–325.28 Mb, respectively. These SNPs could explain 8.95–11.06% and 7.58–21.45% of phenotypic variation, respectively. The significantly correlated loci in the LN condition were mainly distributed on chromosomes 6B and 6D, located at 114.25–116.46 Mb and 460.74–460.79 Mb, which could explain 6.08–6.19% and 6.94–7.82% of phenotypic variation, respectively. Of these significant sites, *AX-109374174* (2A), *AX-110042217* (2A), and *AX-94762593* (6D) appeared in two conditions, and *AX-94875830* (6D) appeared in all three conditions.

In total, 765 significantly related SNPs were detected, including 438, 261, and 408 single nucleotide polymorphisms (SNPs) associated with HN, NN, and LN conditions, respectively (Table S2). It is worth noting that some important QTLs contained more than nine consecutive SNPs that were only detected under a single condition. For example, QTLs on 1B, 3B, and 6D (concentrated in the physical interval of 322.4–325.2 Mb, 521.8–524.5 Mb, and 467.2–471.9 Mb, respectively) were only detected under HN conditions. Only under NN conditions, the QTL composed of 11 significant SNPs located in the physical interval of chromosome 1B (392.7–394 Mb) could be identified. Under LN conditions, a QTL composed of nine significant SNPs controlled both SFW and TDW in the physical range of 59.2–60 Mb located on 2D chromosome, and in the 513.9–518.3 Mb physical interval on chromosome 5A, another QTL composed of 14 significant SNPs that controlled both RFW and TFW was detected.

Moreover, 39 significant SNP loci were found for multiple traits, distributed on all chromosomes except 3A, 3B, 3D, 5A, 5D, and 7D (Table 2). For example, *AX-108962141* (1B) was associated with RDWN, RFWN, SLL, SDWL, SDWN, TDWH, TDWL, and TDWN; *AX-108847203* (1A) and *AX-111170306* (1A) were associated with RDWL, RDWN, SLL, SLN, SDWL, SDWN, TDWL, and TDWN; *AX-109815802* (4B) and *AX-109327593* (4B) exerted pleiotropic effects on RFWN, SFWH, SFWN, TFWH, and TFWN (Table 2).

### 2.6. Haplotype Analysis

**Figure 4:**
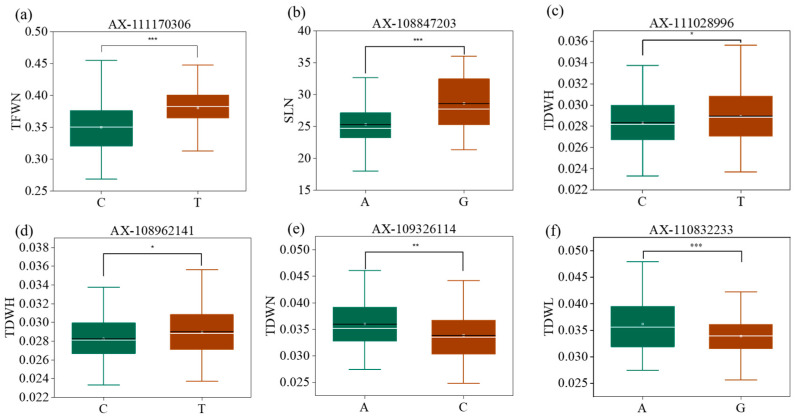
Comparison of the allele effects of SNPs. (**a**) Comparison of the allele effects of *AX-111170306*; (**b**) Comparison of the allele effects of *AX-108847203*; (**c**) Comparison of the allele effects of *AX-111028996*; (**d**) Comparison of the allele effects of *AX-108962141*; (**e**) Comparison of the allele effects of *AX-109336114*; (**f**) Comparison of the allele effects of *AX-110832233*; Trait abbreviations: TFWN, total fresh weight in normal nitrogen; SLN, shoot length in normal nitrogen; TDWH, total dry weight in high nitrogen; TDWN, total dry weight in normal nitrogen; TDWL, total dry weight in low nitrogen. * *p* < 0.05, ** *p* < 0.01, *** *p* < 0.001.

To test the influence of different genotypes on traits, SNPs were identified by GWAS analysis based on BLUP. Furthermore, six SNPs detected in multiple conditions were selected. The population was grouped according to their genotypes and the influence of different genotypes on traits was analyzed using a *t*-test. The results show that the favorable allele of the SNP *AX-111170306* on chromosome 1A increased TFWN from 0.35 to 0.38 (Figure 4a), and from 25.32 to 28.88 for SLN (Figure S4A); the SNP *AX-108847203* increased SLN from 25.33 to 28.61 (Figure 4b) and increased TDWN from 0.034 to 0.038 (Figure S4B). Two SNPs on chromosome 1B, *AX-111028996* and *AX-108962141*, led to TDWN increases of 0.00064 and 0.00075, respectively (Figure 4c,d). The *AX-109326114* SNP on chromosome 1D increased TDWN from 0.034 to 0.036 (Figure 4e) and SFWN from 25.32 to 28.88 (Figure S4C). The *AX-110832233* SNP on chromosome 4D increased TDWL from 0.034 to 0.036 (Figure 4f) and SLL from 21.78 to 23.25 (Figure S4D).

### 2.7. Candidate Genes for Pleiotropic SNPs

We further analyzed the 2 Mb region near the 39 pleiotropic SNPs. According to the Chinese Spring Reference Genome database, 1717 genes were identified (Table S3). The candidate genes were related with antioxidant activity, stimuli-responsive processes (Figure S5A), transport and catabolism, and amino acid metabolism pathways (Figure S5B). For example, the candidate genes *TraesCS1B02G224900*, *TraesCS4D02G361500*, and *TraesCS5B02G039100* encoding nitrate transporter 1 (NRT1) were found near the three SNP loci of *AX-1089962141*, *AX-110832233*, and *AX-111051748*, and *TraesCS2A02G161000* near *AX-11004221* encodes trehalose 6-phosphate, *TraesCS2A02G447200* encodes transcription factor MYB93, and *TraesCS2A02G577000* encodes serine/threonine protein kinase. Two candidate genes on chromosome 2D were related to *AX-111405488*, including *TraesCS2D02G107500* (encoding peroxidase) and *TraesCS2D02G109400* (aspartate protease family); *TraesCS6D02G360800*, a candidate gene related to *AX-94875830* on chromosome 6D, encodes phosphoenolpyruvate carboxylase kinase. The functions of these genes were mainly concentrated on stress resistance, oxidative stress responses, and protein modification pathways. Therefore, we speculate that these genes are involved in nitrogen metabolism and may play a role in nitrogen accumulation (Table S3).

## 3. Discussion

The growth cycle of crops is long and exhibits strong seasonality. In many experiments, the response characteristics of seedling traits to nutrients have been used to reflect the change in the characteristics of whole plant nutrients. Although nitrogen efficiency evaluation at seedling stage is not completely equivalent to nitrogen-use efficiency evaluation at the whole growth stage of wheat, it plays a key role in the growth and development of wheat [39,40]. The hydroponic screening and cultivation period at seedling stage is short and the results are more reliable. Phenotypic data can be obtained easily and quickly by using the existing measuring tools. The identification results have also been shown to be effective in a large number of studies [41,42,43]. Phenotypic data of hydroponics at seedling stage can be used as an important reference standard for evaluating nitrogen use efficiency [44,45].

The LD attenuation distance in a population determines the marker coverage density required for GWAS. In this study, the whole genome LD decay distance of 3.5 Mb was reported: 4.6 Mb for the A genome, 3.5 Mb for the B genome, and 2.5 Mb for the D genome (Figure 3c). Similar LD patterns have been reported previously [46,47].

### 3.1. Phenotypic Analysis of Wheat under Different Conditions

The root system is important for supporting the development of the aboveground part of the plant; it directly detects nutrient signals and absorbs nutrients, and the survival and performance of the crop largely depend on the plant’s ability to absorb nutrients [48]. Studies show a positive correlation between panicle number and root development in wheat [49]. Wheat has a large number of branches in the middle soil (20–70 cm) with strong growth, and its root length and number densities are also high [50]. Strongly growing wheat has a high nitrogen uptake rate, which could reflect the characteristics of nitrogen uptake and utilization in wheat to some extent [51]. In this study, there were obvious differences in root configuration under different culture conditions. The mean RL in the LN condition was significantly longer than that in the NN and HN conditions, and the RL in the HN condition was thin with numerous lateral roots, suggesting that excessive nutrition might inhibit the growth of seedling roots. Excess nitrogen can lead to delayed plant growth and increased susceptibility to diseases and insect pests.

### 3.2. Analysis Related Loci of Wheat Seedling Traits

Many QTLs have been detected in wheat treated with different nitrogen levels [52,53,54]. However, most known QTLs were identified under two nitrogen conditions. The correlation analysis of nine seedling traits of wheat was carried out under three conditions of HN, NN, and LN. Based on the common phenomenon of overapplication of nitrogen fertilizer in wheat production, using three nitrogen conditions can help better explain the response of wheat to nitrogen. Phenotypic data were analyzed with BLUP values to obtain more accurate results [55].

In this study, certain SNP loci were located close to known loci associated with traits. For example, in the LN and NN conditions, a polymorphic SNP locus on chromosome 4D controlled the length of the aboveground portion (*AX-89398511*; 17 Mb); this is in the same region as the SNP locus *QPh-4D* (16–19 Mb) that controlled plant height in previous studies and may, therefore, be the same QTL site. *AX-110026721*, identified at 34.32 Mb on chromosome 2A, is 2 Mb apart from the locus at 30.9–32.0 Mb on chromosome 2A [56]. Three SNPs on chromosome 2B are associated with TDW, SDW, and TFW (*AX-109621732*, *AX-111672733*, and *AX-94559451*); these are 0.38, 0.000035, and 1.34 Mb away from *Affx-109177363*, *Affx*-*109254520*, and *Affx-88661145*, which were associated with TDW, SDW, and TFW in a previous study [57]. In the HN condition, the distance between the SNP on chromosome 3B for RFW (*AX-109517098*) and the previous SNP for RFW under normal moisture conditions (*AX-109055035*) was approximately 0.73 Mb. In the HN condition, *AX-109741930* and *AX-108966945* on chromosome 6D for RDW were 0.97 and 0.019 Mb away from *AX-110986216* and *AX-108905447* for RDW, respectively [57].

Meanwhile, we identified some possible new QTLs within our known range; some important QTLs contained more than nine consecutive SNPs were only associated under a single condition, such as QTLs related to RSR, RFW, and RDW on 1B (322.4–325.2 Mb), 3B (521.8–524.5 Mb), and 6D (467.2–471.9 Mb) under HN condition, QTL sited on 1B (392.7–394 Mb) under NN condition, and QTLs correlated with both SFW and TDW traits on 2D (59.2–60 Mb) and 5A (513.9–518.3 Mb) under LN condition. These results indicate that these QTLs are affected by nitrogen levels and belong to nitrogen-sensitive QTL sites. Therefore, these QTLs have certain reference value for the subsequent marker-assisted selection of wheat varieties with high efficiency. Five SNPs (*AX-108876381*, *AX-108962141*, *AX-110832233*, *AX-111028996*, and *AX-94875830*) were detected in all conditions when controlling for the same trait. These SNPS are more adaptable to N conditions, and further studies on these important QTLs and SNPs will be conducted in the future.

### 3.3. Prediction of Relevant Candidate Genes

Based on the reference genome IWGSC V1.1, a total of 1717 genes were identified in functional annotation performed of related genes near SNP sites with multiple effects; these encoded proteins and enzymes are involved in transporters, hydrolases, peroxidases, glycosyltransferases, oxidoreductases, acyltransferases, ubiquitin ligases, and sucrose synthetases.

In *Arabidopsis thaliana*, NRT1.1 in the NRT1 family is the parent and transporter, and its expression can be induced by both high and low NO_3_^−^ concentrations [58]. NRT1.2 is mainly expressed in root epidermal cells and participates in NO_3_^−^ transport [59]. NRT1.9 is expressed in the root primitive cortex and plays an important role in nitrogen transport [60]. NRT1.5 is involved in long-distance transport from root to stem [61]. NRT1.8 is mainly involved in the transport of NO_3_^−^ in xylem [62]. NRT1.7 is mainly expressed in the phloem of old leaves, involved in the transport of NO_3_^−^ from old leaves to young leaves, and controls the transport of NO_3_^−^ in the phloem of leaves [63]. Candidate genes *TraesCS1B02G224900*, *TraesCS4D02G361500,* and *TraesCS5B02G039100* encode NRT1. It is speculated that the above three candidate genes are related to NO_3_^−^ transport, although some NRT1 genes have been found in crops such as wheat, rice, and maize [64,65]. However, there are few studies on the function and expression sites of this gene family, and further exploration, identification, and expression are needed.

Park et al. noted that E3 ubiquitin protein ligase is essential for primary root growth and bud development [66,67]. The E3 ubiquitin ligase LOG2 is involved in regulating amino acid export from plant cells, which is the main method of transporting organic N in xylem and phloem [68,69]. For example, Min et al. proposed that the E3 ubiquitin ligase CaPUB1 could enhance cold resistance in transgenic rice [70]. *TraesCS4B02G016000*, *TraesCS6D02G360000*, *TraesCS4D02G356400*, *TraesCS4D02G362000*, *TraesCS6A02G096200*, *TraesCS2A02G448700*, *TraesCS2A02G178500*, and *TraesCS4D02G357700* are E3 ubiquitin protein ligases, which may be regulated by N. The candidate genes *TraesCS6D02G356600*, *TraesCS2D02G109400*, *TraesCS2D02G109500*, *TraesCS2D02G109600*, *TraesCS1B02G414800*, *TraesCS7A02G088300*, *TraesCS7A02G088400*, and *TraesCS5B02G038800* encode aspartic proteases that are involved in protein processing and degradation during plant growth and development. Under biological and abiotic stresses, the expression level of the aspartic protease gene *FeAP9* is upregulated. *TraesCS6A02G076300*, *TraesCS6D02G358600*, *TraesCS4A02G108700*, and *TraesCS2A02G577400* are associated with serine/threonine protein kinases and promote protein phosphorylation through broad regulatory mechanisms. These factors include glucose metabolism, photosynthesis, and maladaptive environment. These genes identified by screening may affect the basic nitrogen metabolism process of plants and, thus, play an important role in crop nitrogen utilization.

## 4. Materials and Methods

### 4.1. Plant Material

A total of 204 species of bread wheat were used for this GWAS. Among them, 168 were Chinese wheat germplasm resources from Henan (21), Hebei (33), Sichuan (36), Yunnan (27), Xinjiang (25), and other important wheat growing areas. A total of 36 were imported varieties, from the United States, France, Italy, Mexico, and other countries. The panel possesses high genetic diversity of wheat breeding (Table S1).

### 4.2. Experimental Design

The paper roll culture method [71] was used to cultivate and process the research materials. The experiment included three treatments: low nitrogen (LN; 0.1 mM), normal nitrogen (NN; 5 mM), and high nitrogen (H; 45 mM). Ammonium nitrate was the only source of nitrogen and the other nutrient solution compositions were consistent with those of the standard Hoagland nutrient solution. Fifty wheat seeds of similar sizes were selected, soaked in 10% H_2_O_2_ for 20 min, washed with distilled water, soaked in saturated CaSO_4_ for 6 h to disinfect the seeds, and washed under flowing distilled water until the seeds germinated. Germination paper from Anchor Company, USA was sterilized at 121 °C for 20 min to prevent fungal growth during seedling development. Each soaked wheat seed was placed upside down approximately 1.5 cm from the edge of the blotting paper, spaced approximately 1.5 cm from the next wheat seed. In total, 12 seeds of each variety were tested for each treatment. The seeds were covered with another sheet of blotting paper and rolled up from one side to the other. The rolled wheat seeds were placed in different hydroponic nutrient solutions for each treatment in an incubator with the following conditions: photoperiod, 14/10 h (light/dark); relative humidity, 45%; temperature, 25 °C; and light intensity, 8600 Lux. The nutrient solution was replaced every two days to maintain cleanliness and stability. After culturing for 15 days, all traits were determined. The experiment was repeated four times.

### 4.3. Phenotype Measurement and Data Analysis

The root length (RL, cm) and shoot length (SL, cm) of the 12 seedlings were measured manually. Plants were divided into shoots and roots. Excess water was removed with absorbent paper before weighing with a 1/10,000 balance to obtain the root fresh weight (RFW, g) and shoot fresh weight (SFW, g). Each plant was killed at 105 °C for 30 min and dried to a constant weight at 80 °C. The root dry weight (RDW, g) and shoot dry weight (SDW, g) were determined. The root–shoot ratio (RSR), total dry weight (TDW, g), and total fresh weight (TFW, g) were calculated.

Variance analysis was performed on the phenotypic data of the nine traits under treatment with different nitrogen concentrations using SPSS software (version 21.0; IBM Inc., Armonk, NY, USA). Using the R software (version 4.3.0; Vienna, Austria) package “lme4”, optimal linear unbiased prediction (BLUP) values were obtained for each trait from the four repetitions, and these predictors were later used for GWAS analysis. The formula below was used to calculate the generalized heritability (*h*^2^) of each trait.

The formula:

*h*^2^ = *V_G_*/(*V_G_* + *V_E_*), (1)

where *V_G_* represents genetic variance and *V_E_* represents environmental variance.

### 4.4. Population Structure and Linkage Disequilibrium Analysis

The total genomic DNA of young wheat leaves was extracted using the improved cetyltrimethylammonium bromide (CTAB) method [72]. After quality inspection, 204 wheat species were genotyped using a 55 K wheat iSelect SNP array by China Golden Marker Biotechnology Co., Ltd. (Beijing, China). A total of 53,007 SNP loci were obtained from 204 wheat genotypes using the 55 K Wheat iSelect SNP array. After filtering (missing rate > 10% and minor allele frequency < 5%), 32,008 SNPs were used for the structure, LD and neighbor-joining tree. Power Marker V3.25 software was used to analyze the genetic diversity and polymorphism information content (PIC) of the test materials:

PIC = 1 − ∑(P*ij*)^2^, (2)

where P*ij* indicates the frequency of the allelic variation *j* at the site *i*.

The population structure of the panel was inferred using the STRUCTURE 2.3.4 software [73] for each K (K = 2–8). Linkage disequilibrium (LD) analysis of the whole genome and chromosomes A, B, and D of bread wheat was performed using the marker data screened in TASSEL 5.2.82. The LD decay rate was measured as the physical distance at which the average pairwise r^2^ dropped to half its maximum value [31]. The LD decay distance was obtained by constructing a scatter plot of r^2^ values and SNP pair distances and fitting these points to a smooth curve using Origin software (version 2021; OriginLab, Northampton, MA, USA). *p* = 0.001 was set as the threshold for all conditions. A neighbor-joining tree was constructed using the TASSEL 5.0 (version 5.2.82; Buckler Laboratory, Cornell University) [74].

### 4.5. Genome-Wide Association Analysis and Candidate Gene Analysis

In order to avoid too many false positives in the results of association analysis, the affinities of the tested natural population were taken as covariates in the subsequent association analysis [75]. The calculation was performed using the mixed linear model (MLM) (Q + K) in TASSEL software. A suggestive threshold *p*-value of 0.001 was used to estimate significant SNPs [76,77]. The “CMplot” package in R was used to draw Manhattan plots.

Stable SNPs in the four experiments were selected for favorable allele analysis. In the analysis of allele effects on each trait, alleles with positive effects leading to higher trait values were described as “favorable alleles,” whereas those leading to lower values were “unfavorable alleles” [78]. Based on these SNP sites, the tested species were divided into two haplotypes and a *t*-test was used to detect and compare the significance of the differences in the target traits between the two haplotypes. Origin 2021 was used to generate a boxplot for haplotype analysis. The R package “CMPlot” was used to produce a Manhattan map and to mine candidate genes for wheat traits within the LD attenuation range of these SNP sites. The SNP sequence was used to search the Chinese Spring Wheat Genome Database and obtain the interval sequence. The BLAST sequence was compared in the NCBI database. Gene function was annotated using Gene Ontology (http://www.geneontology.org, accessed on 1 August 2023) and the Kyoto Encyclopedia of Genes and Genomes pathway database (http://www.genome.jp/kegg, accessed on 1 August 2023). To investigate potential candidate genes, the Ensembl Plants database (http://plants.ensembl.org, accessed on 1 August 2023) was used to download and function query the nearby genes of significant SNPs under the three conditions [77].

## 5. Conclusions

In this study, 204 wheat accessions were evaluated related traits under low-, normal, and high-nitrogen conditions and were genotyped using Wheat55K SNP array. GWAS analysis shows that a total of 765 SNPs was significantly associated with nine traits in wheat under three different nitrogen conditions. Additionally, there are three, one, and two important QTL loci found to be associated only under HN, NN, and LN conditions, respectively. The resulting data were further analyzed to identify relevant SNP loci and candidate genes, which can be used to breed new wheat species with relatively low nitrogen fertilizer requirements and high efficiency by using marker-assisted selection in the future to achieve efficient conservation and utilization of nitrogen and nitrogen resources.

## Figures and Tables

**Table 1:** Statistics for nine root and seedling biomass traits in the association population based on BLUP values.

Conditions	Index	RL	RSR	RDW	RFW	SL	SDW	SFW	TDW	TFW
HN	Min	13.14	0.17	0.004	0.077	16.82	0.020	0.144	0.022	0.227
	Max	15.30	0.39	0.007	0.109	24.97	0.027	0.170	0.038	0.264
	Ave	14.17	0.22	0.005	0.089	19.83	0.024	0.157	0.029	0.246
	SD	0.36	0.03	0.001	0.005	1.51	0.001	0.005	0.003	0.008
	Coef. of var (%)	2.56	12.15	7.130	5.272	7.62	5.850	2.987	9.666	3.122
	*h* ^2^	0.92	0.47	0.470	0.774	0.57	0.381	0.860	0.398	0.618
NN	Min	12.76	0.21	0.005	0.077	18.02	0.021	0.161	0.015	0.233
	Max	19.17	0.32	0.009	0.159	36.03	0.050	0.305	0.064	0.455
	Ave	16.02	0.26	0.007	0.117	25.74	0.035	0.237	0.035	0.354
	SD	1.09	0.02	0.001	0.014	3.43	0.005	0.027	0.009	0.041
	Coef. of var (%)	6.76	7.93	11.299	12.161	13.34	14.268	11.519	24.837	11.44
	*h* ^2^	0.79	0.72	0.684	0.597	0.40	0.777	0.673	0.327	0.620
LN	Min	14.33	0.34	0.007	0.113	17.69	0.016	0.135	0.023	0.251
	Max	20.59	0.66	0.013	0.206	30.78	0.037	0.229	0.048	0.434
	Ave	17.58	0.44	0.010	0.154	22.27	0.024	0.185	0.035	0.339
	SD	1.12	0.04	0.001	0.016	2.16	0.004	0.017	0.004	0.031
	Coef. of var (%)	6.39	9.49	9.097	10.065	9.70	14.362	9.341	11.671	9.044
	*h* ^2^	0.92	0.62	0.698	0.473	0.59	0.135	0.450	0.288	0.467

Trait abbreviations: RL, root length; RSR, root–shoot ratio; RDW, root dry weight; RFW, root fresh weight; SL, shoot length; SDW, shoot dry weight; SFW, shoot fresh weight; TDW, total dry weight; TFW, total fresh weight; HN, high nitrogen; NN, normal nitrogen; LN, Low nitrogen; SD, standard deviation; *h*^2^, broad-sense heritability.

**Table 2:** The SNP significantly associated with pleiotropic effect in different phenotyping traits in two environments.

Marker	Chromosome	Position	Pleiotropic Effect	SNP	*p*-Value
*AX-108847203*	1A	567714120	RDWL, RDWN, SLL, SLN, SDWL, SDWN, TDWL, TDWN	G	<0.001
*AX-111170306*	1A	567967912	RDWN, SLL, SLN, SDWL, SDWN, SFWN, TDWL, TDWN, TFWN	T	<0.001
*AX-108737478*	1B	375098459	TDWL, TFWN	C	<0.001
*AX-111695833*	1B	374522868	RDWL, TDWH	A	<0.001
*AX-108962141*	1B	403813869	RDWN, RFWN, SLL, SDWL, SDWN, TDWH, TDWL, TDWN	T	<0.001
*AX-110392069*	1B	639638582	SDWN, SFWN, TDWH, TDWN, TFWN	G	<0.001
*AX-109326114*	1D	21743856	RDWN, SLL, SDWL, SDWN, SFWN, TDWN	A	<0.001
*AX-111161220*	1B	414806318	RDWN, TDWH	T	<0.001
*AX-108903381*	1B	415260486	RSRH, SFWN	A	<0.001
*AX-108786044*	2A	698009743	RDWL, SFWH, TDWH, TDWL, TFWH	A	<0.001
*AX-110362294*	1B	317448975	RSRH, SFWN, TFWN	T	<0.001
*AX-111575379*	2B	461886409	RDWN, TDWH, TDWL	A	<0.001
*AX-111672733*	2B	572628142	SDWL, TDWH, TDWL	C	<0.001
*AX-110010230*	1B	325283703	RSRH, SFWN, TFWN	C	<0.001
*AX-111405488*	2D	59943039	SDWL, SFWL, TDWH, TDWL	C	<0.001
*AX-110926323*	1B	325256496	RSRH, SFWN	T	<0.001
*AX-110438187*	2A	95830292	RSRH, SLN	A	<0.001
*AX-109815802*	4B	10916305	RFWN, SFWH, SFWN, TFWH, TFWN	A	<0.001
*AX-109327593*	4B	10944746	RFWN, SFWH, SFWN, TDWH, TFWN	A	<0.001
*AX-109365636*	2B	758808487	SDWN, SFWN, TDWH, TFWN	T	<0.001
*AX-110832233*	4D	507128999	SLL, SLN, SDWL, SDWN, TDWH, TDWL, TDWN	A	<0.001
*AX-111051748*	5B	44358312	SDWL, TDWH, TDWL	A	<0.001
*AX-89696347*	2D	647533340	RLH, SDWL, TDWL	C	<0.001
*AX-109325100*	6A	61431458	SDWN, SFWN, TDWH, TDWN, TFWN	A	<0.001
*AX-111342539*	2D	100544989	TDWH, TFWN	A	<0.001
*AX-109651481*	6A	310569613	SDWL, SFWL, TDWH, TDWL, TFWL	C	<0.001
*AX-108871687*	6B	553420734	RDWL, RDWN, TDWH, TDWL	G	<0.001
*AX-111605442*	2D	220630228	TDWH, TFWN	C	<0.001
*AX-110525380*	3B	25322853	RFWH, TFWH	T	<0.001
*AX-111123066*	7A	55431597	SDWN, SFWN, TDWH, TDWN, TFWN	T	<0.001
*AX-111526214*	4D	310458135	SFWN, TDWH, TFWN	C	<0.001
*AX-110483224*	7B	611776655	SLL, SLN, SDWN	C	<0.001
*AX-109327847*	7B	612256762	SLL, SLN, SDWN, SFWN	A	<0.001
*AX-111485326*	7B	627697145	SDWN, TDWL, TDWN	A	<0.001
*AX-109401644*	5B	50655258	SDWN, TDWN	A	<0.001
*AX-111721212*	5D	553775393	RDWL, RFWN, TDWL	C	<0.001
*AX-111762061*	6B	192866992	RFWN, SDWN, SFWN, TDWH, TFWN	T	<0.001
*AX-109624261*	7A	113627131	SFWN, TDWH, TFWN	G	<0.001
*AX-110595073*	7B	627636405	SLL, SDWN, TDWN	A	<0.001

## Data Availability

The data presented in this study are available within the article and its Supplementary Materials.

## Supplementary Materials

The following supporting information can be downloaded at: https://www.mdpi.com/article/10.3390/plants12234050/s1, Table S1: Variety name and origin of 204 wheat materials; Table S2: The SNPs significantly associated with nine root and seedling bioNass traits for each environNent based on BLUP values. Trait abbreviation: RLH, root length in high nitrogen environNent; Table S3: The 1717 genes inside the 2 Mb region on each side on 41 SNPs stable among chromosomes in three environments; Figure S1. Manhattan plots for the three root and seedling biomass traits identified by genome-wide association study (GWAS) using BLUP values. The dashed line represents the significance threshold (−log10 P = 3.0). Trait abbreviations: (A) RLH, root length in high nitrogen; RLN, root length in normal nitrogen; RLL, root length in low nitrogen, (B) RFWH, root fresh weight in high nitrogen; RFWN, root fresh weight in normal nitrogen; RFWL, root fresh weight in low nitrogen, (C) RDWH, root dry weight in high nitrogen; RDWN, root dry weight in normal nitrogen; RDWL, root dry weight in low nitrogen; Figure S2. Manhattan plots for the three root and seedling biomass traits identified by genome-wide association study (GWAS) using BLUP values. The dashed line represents the significance threshold (−log10 P = 3.0). Trait abbreviations: (A) SLH, shoot length in high nitrogen; SLN, shoot length in normal nitrogen; SLL, shoot length in low nitrogen, (B) SFWH, shoot fresh weight in high nitrogen; SFWN, shoot fresh weight in normal nitrogen; SFWL, shoot fresh weight in low nitrogen, (C) SDWH, shoot dry weight in high nitrogen; SDWN, shoot dry weight in normal nitrogen; SDWL, shoot dry weight in low nitrogen; Figure S3. Manhattan plots for the three root and seedling biomass traits identified by genome-wide association study (GWAS) using BLUP values. The dashed line represents the significance threshold (−log10 P = 3.0). Trait abbreviations: (A) RSRH, root-shoot ratio in high nitrogen; RSRN, root-shoot ratio in normal nitrogen; RSRL, root-shoot ratio in low nitrogen, (B) TFWH, total fresh weight in high nitrogen; TFWN, total fresh weight in normal nitrogen; TFWL, total fresh weight in low nitrogen, (C) TDWH, total dry weight in high nitrogen; TDWN, total dry weight in normal nitrogen; TDWL, total dry weight in low nitrogen; Figure S4: Comparison of the allele effects of SNPs. Trait abbreviations: (A) SLN, shoot length in normal nitrogen, (B) TDWN, total dry weight in normal nitrogen, (C) SFWN, shoot fresh weight in normal nitrogen, (D) SLL, shoot length in low nitrogen. * *p* < 0.05, *** *p* < 0.001; Figure S5: (A) GO annotation analysis of the candidate genes; (B) KEGG annotation analysis of the candidate genes.
