# Supplementary material for: Genome-Wide Association Study on Seedling Phenotypic Traits of Wheat under Different Nitrogen Conditions
Source: Plants (Basel). 2023 Dec 1;12(23):4050. doi: 10.3390/plants12234050 (PMC10707948; doi:10.3390/plants12234050)
Supplement: Supplementary file 1 [file plants-12-04050-s001.zip › Supplementary Marterial.pdf]

Figures

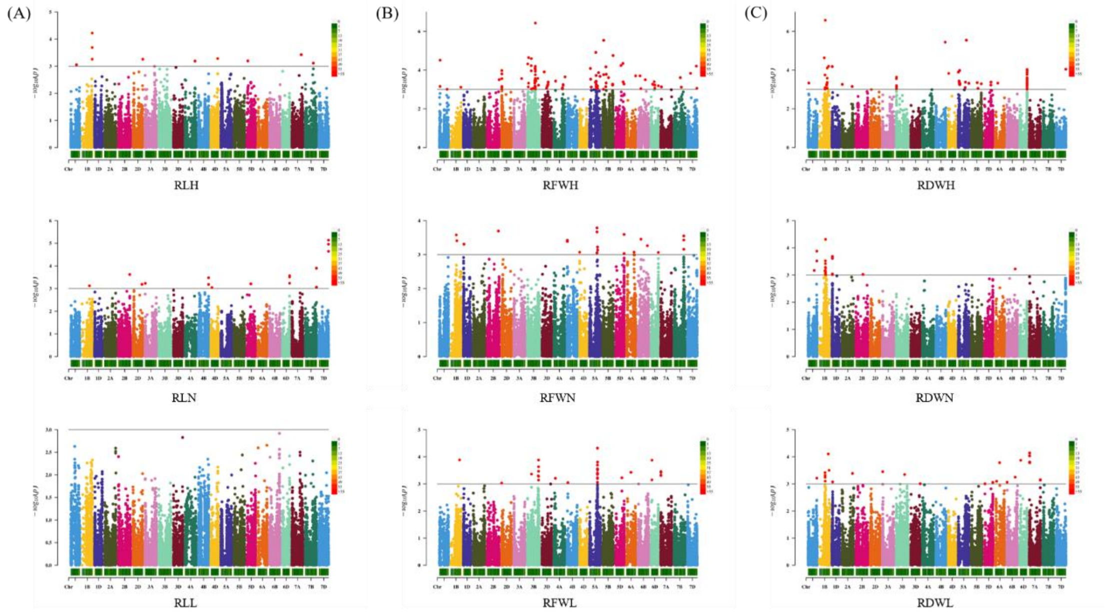

Figure S1. Manhattan plots for the three root and seedling biomass traits identified by genome-wide association study (GWAS) using BLUP values. The dashed line represents the significance threshold (–log10 *P* = 3.0). Trait abbreviations: **(A)** RLH, root length in high nitrogen; RLN, root length in normal nitrogen; RLL, root length in low nitrogen; **(B)** RFWH, root fresh weight in high nitrogen; RFWN, root fresh weight in normal nitrogen; RFWL, root fresh weight in low nitrogen; **(C)** RDWH, root dry weight in high nitrogen; RDWN, root dry weight in normal nitrogen; RDWL, root dry weight in low nitrogen.

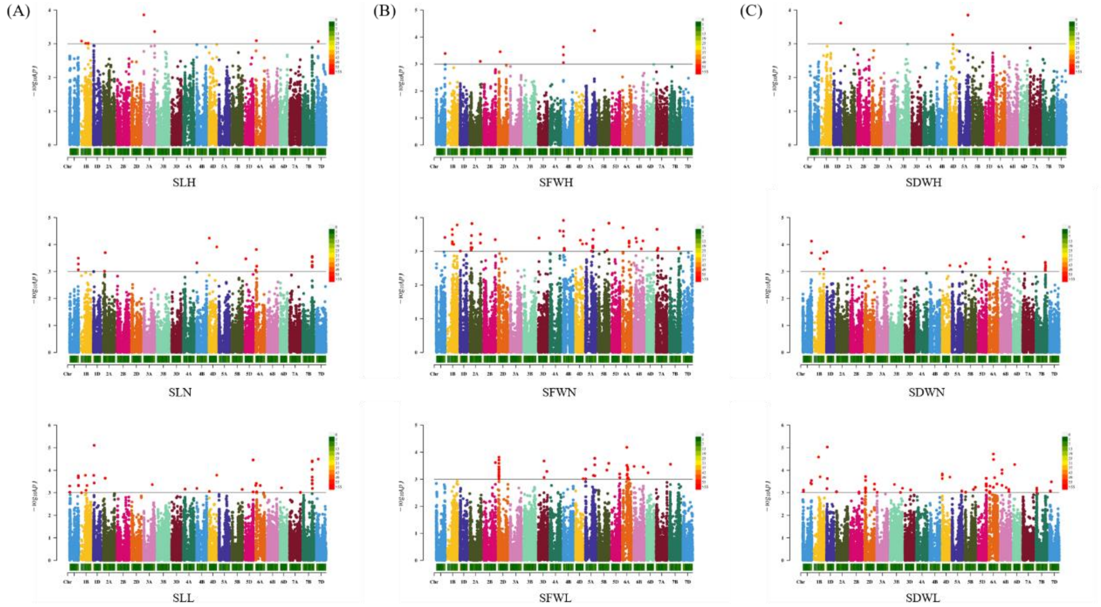

Figure S2. Manhattan plots for the three root and seedling biomass traits identified by genome-wide association study (GWAS) using BLUP values. The dashed line represents the significance threshold (–log10 *P* = 3.0). Trait abbreviations: **(A)** SLH, shoot length in high nitrogen; SLN, shoot length in normal nitrogen; SLL, shoot length in low nitrogen; **(B)** SFWH, shoot fresh weight in high nitrogen; SFWN, shoot fresh weight in normal nitrogen; SFWL, shoot fresh weight in low nitrogen; **(C)** SDWH, shoot dry weight in high nitrogen; SDWN, shoot dry weight in normal nitrogen; SDWL, shoot dry weight in low nitrogen.

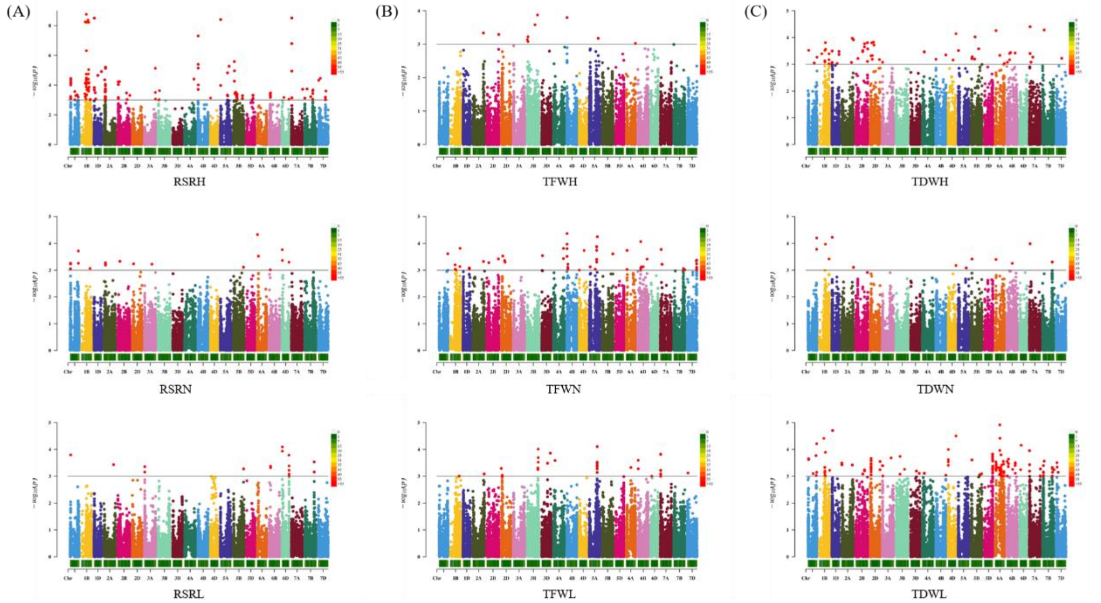

Figure S3. Manhattan plots for the three root and seedling biomass traits identified by genome-wide association study (GWAS) using BLUP values. The dashed line represents the significance threshold (–log10 *P* = 3.0). Trait abbreviations: **(A)** RSRH, root-shoot ratio in high nitrogen; RSRN, root-shoot ratio in normal nitrogen; RSRL, root-shoot ratio in low nitrogen, **(B)** TFWH,

total fresh weight in high nitrogen; TFWN, total fresh weight in normal nitrogen; TFWL, total fresh weight in low nitrogen; **(C)** TDWH, total dry weight in high nitrogen; TDWN, total dry weight in normal nitrogen; TDWL, total dry weight in low nitrogen.

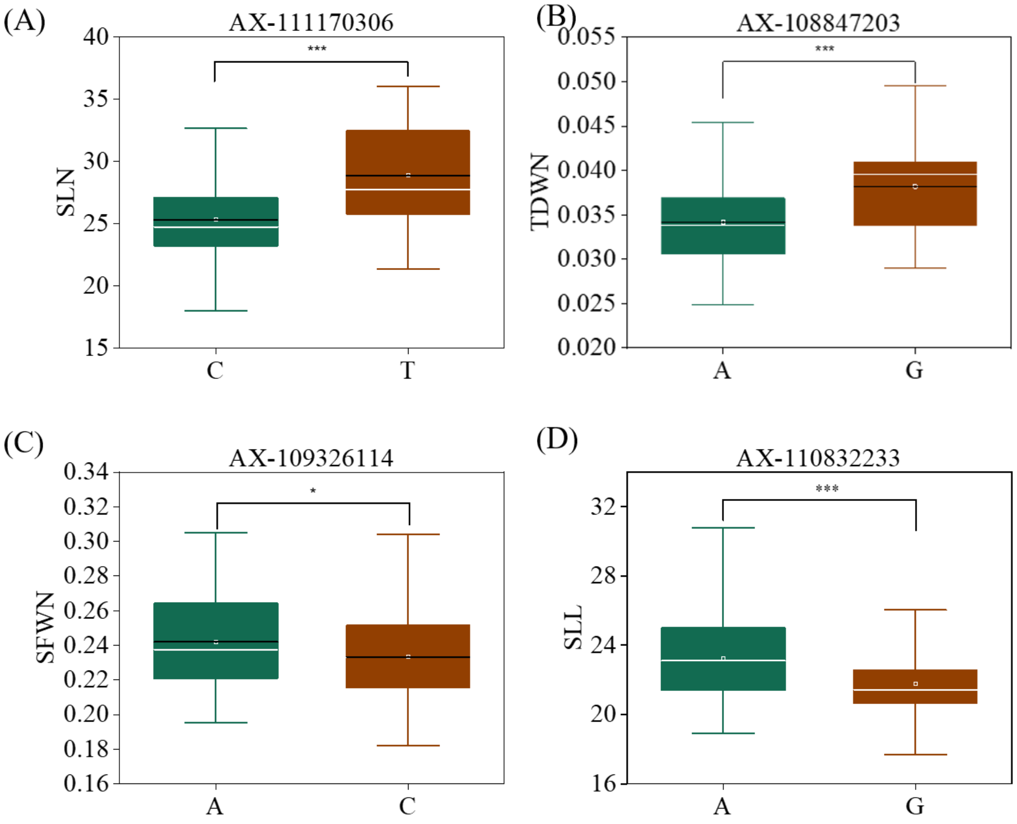

Figure S4. Comparison of the allele effects of SNPs. Trait abbreviations: **(A)** SLN, shoot length in normal nitrogen; **(B)** TDWN, total dry weight in normal nitrogen; **(C)** SFWN, shoot fresh weight in normal nitrogen; **(D)** SLL, shoot length in low nitrogen. *$p < 0.05$, ***$p < 0.001$.

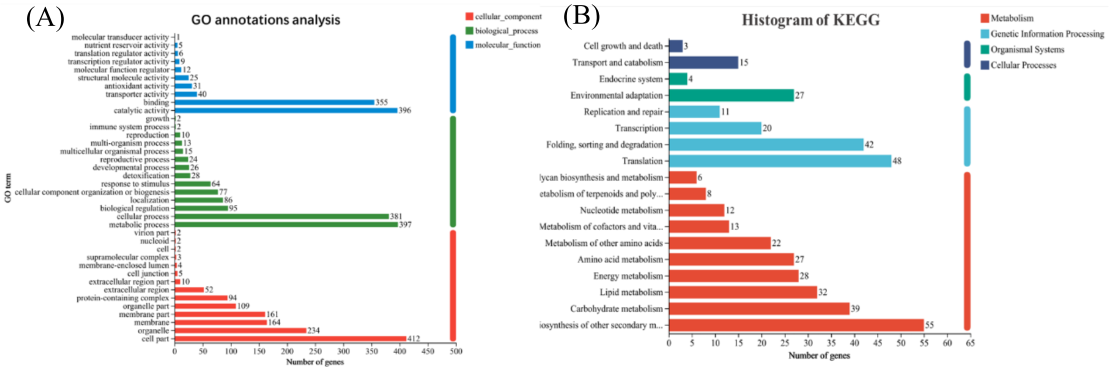

Figure S5. GO and KEGG annotation analysis of candidate genes.**(A)** GO annotation analysis of the candidate genes; **(B)** KEGG annotation analysis of the candidate genes.
